# “Life-like” assessment of antimicrobial surfaces by a new touch transfer assay displays strong superiority of a copper alloy compared to silver containing surfaces

**DOI:** 10.1371/journal.pone.0187442

**Published:** 2017-11-14

**Authors:** Johannes Karl-Mark Knobloch, Sabrina Tofern, Wladimir Kunz, Sara Schütze, Michael Riecke, Werner Solbach, Thomas Wuske

**Affiliations:** 1 University Medical Center Hamburg-Eppendorf, Institute for Medical Microbiology, Virology and Hygiene, Department of Hospital Hygiene, Hamburg, Germany; 2 German Center for Infection Research, Partner site Hamburg-Lübeck-Borstel, Germany; 3 University Hospital of Schleswig-Holstein, Campus Lübeck, Clinic for Medical Microbiology and Infectiology, Lübeck, Germany; 4 Drägerwerk AG & Co. KGaA, Lübeck, Germany; 5 University Lübeck, Center for Infection and Inflammation Research (ZIEL), Lübeck, Germany; VIT University, INDIA

## Abstract

Transmission of bacteria from inanimate surfaces in healthcare associated environments is an important source of hospital acquired infections. A number of commercially available medical devices promise to fulfill antibacterial activity to reduce environmental contamination. In this study we developed a touch transfer assay modeling fingerprint transmission to investigate the antibacterial activity of surfaces, with confirmed antibacterial activity by a modified ISO 22196 (JIS Z 2801) assay to test such surfaces under more realistic conditions. Bacteria were taken up from a dry standardized primary contaminated surface (PCS) with disinfected fingers or fingers covered with sterile and moistened cotton gloves. Subsequently, bacteria were transferred by pressing on secondary contaminated surfaces (SCS) with or without potential antibacterial activity and the relative reduction rate was determined after 24 h. A stable transmission rate between PCS and SCS was observed using moistened sterile gloves. A copper containing alloy displayed at least a tenfold reduction of the bacterial load consistently reaching less than 2.5 cfu/cm^2^. In contrast, no significant reduction of bacterial contamination by silver containing surfaces and matured pure silver was observed in the touch transfer assay. With the touch transfer assay we successfully established a new reproducible method modeling cross contamination. Using the new method we were able to demonstrate that several surfaces with confirmed antimicrobial activity in a modified ISO 22196 (JIS Z 2801) assay lacked effectiveness under defined ambient conditions. This data indicate that liquid based assays like the ISO 22196 should be critically reviewed before claiming antibacterial activity for surfaces in the setting of contamination of dry surfaces by contact to the human skin. We suggest the newly developed touch transfer assay as a new additional tool for the assessment of potential antimicrobial surfaces prior utilization in hospital environments.

## Introduction

Healthcare-acquired infections (HAI) cause substantial patient morbidity and mortality [1]. For some organisms it is widely accepted that inanimate surfaces can contribute to the transmission of pathogens within hospitals [2]. Especially pathogens which are able to survive on surfaces for weeks, play an important role as a reservoir for HAI. Thereby, infection might occur following cross-contamination via patient or surface contact [3;4]. Studies indicate that pathogens as MRSA, VRE and *Acinetobacter baumannii* are shed from patients and spread by patients or health care workers to surfaces via touch transfer processes in the immediate vicinity of the patient [5]. Rooms with a history of colonized or infected patients have been suspected to be the source for infection of subsequent patients despite of disinfection measures [6].

Therefore, reduction in surface bioburden is required to reduce HAIs. As a measure to improve surface hygiene, quality standards for disinfection and microbiological surface screening have been proposed to break the nosocomial infection loop with a suggested threshold of 2.5 cfu/cm^2^, proposed as a microbiologic standard for safer hospital environments [7;8]. The use of biocidal surface materials in conjunction with improved disinfection and hygiene protocols could eliminate bioburden, rather than relying solely on surface cleaning agents or irradiation methods. In consequence, the development of antimicrobial surface coatings has been designed as a reinforcement measure to tackle surface contaminations between cleanings. Apart from antifouling and anti-adhesive materials as preventative coatings, antimicrobial reagents have been implemented to plastics, varnishes and paints or alloys as recently reviewed [9–11].

Several methods are described to investigate potential antimicrobial activity of surfaces. For solid surfaces results from assays following the ASTM E 2180 [12] standard or the ISO 22196 standard [13], which is a modification of the Japanese Standard JIS Z2801 are used as base for suppliers of health care equipment to claim antimicrobial activity. In contrast to a usual hospital environment with dry surfaces around patients and comfort humidity and temperature of the air these standardized methods use test conditions with maintaining significant amounts of liquid to allow diffusion of antimicrobial compounds as well as temperatures up to 37°C. This, however, does not mirror reality. Therefore, these methods might fail to predict the efficacy of antibacterial surfaces under realistic conditions.

For surfaces manufactured from copper or copper-alloys alternative test methods were developed, which address dry conditions during incubation [14–16]. However, in the US Environmental Protection Agency (EPA) assays the primary inocula are still liquid bacterial solutions with varying drying times prior reaching dry conditions which still does not reflect the typical contamination of high touch surfaces by the human skin [17;18].

In this study, we have analyzed the typical contamination of high touch surfaces with frequent contact to human skin to define commonly observed contaminating settings. Subsequently we developed a new, reproducible method modeling cross contamination caused by touch transfer referring to the typical contamination of high touch surfaces. Using this new method we have analyzed several surfaces confirmed to have antimicrobial activity according to prior standardized testing using a modified ISO 22196 assay.

## Materials and methods

### Environmental sampling from space bars

To estimate environmental contamination of high touch surfaces 47 space bars of computer keyboards on hospital wards (n = 24) and outside the hospital (n = 23) were investigated by quantitative culture. Bacteria were removed from the space bars by heavy scrubbing with a moistened Ʃ-Transwab (Medical Wire, Corsham, UK) with subsequent transfer in 1mL liquid Amies medium. 100 μl of the suspensions were streaked on Columbia sheep blood agar (COS) plates (bioMérieux, Marcy l’Etoile, France) using Drigalski spatula and bacterial counts were determined after 24 h at 36°C ± 2°C and total viable counts were calculated (detailed results are presented as supporting information).

### Used surfaces and preparation of surfaces

As surfaces for primary contamination and as secondary surfaces for quantitative experiments during the evaluation of the touch transfer assay standardized 5 x 5 cm ceramic tiles with white matt glaze (#3709PN00, Villeroy & Boch, Mettlach, Germany) were used. Films promising antibacterial activity (HEXIS-Health, Hilden, Germany; iShieldz, eShields, La Verne, CA, USA) were placed on 5 x 5 cm stainless steel UNS S30400 plates. For the investigation of pure silver or copper alloy 5 x 5 cm plates were purchased from the manufacturer (99,9% silver, Jeddeloh, Germany; KME Plus^®^ coolsilver copper alloy, KME, Germany; CuNi30Mn1Fe, corresponding to the Unified Numbering System [UNS] for copper + copper alloys C71500 or former C71630). To get rid of oxidized upper layers, metals were sanded down with 600 grade grit-sandpaper, if necessary. All used surfaces were sterilized using 70% isopropyl alcohol prior to experimental sets.

### Modified ISO 22196 (Japanese Industrial Standard Z 2801) test

Testing of antibacterial activity of all surfaces was performed using a slightly modified ISO 22196 (JIS Z 2801) test as followed. *S*. *aureus* ATCC 33591 was cultivated on COS agar at 36°C ± 2°C overnight and inoculated in sterile 0,85% NaCl solution to reach bacterial solutions with a density of 5 x 10^5^ colony forming units (cfu)/mL. Sterilized surfaces were inoculated with 400 μL of this suspension and were covered by sterilized 4 x 4 cm stomacher bag film. The covered surfaces were incubated at 36°C ± 2°C with a relative humidity of ≥90% for 24 h. As a control bacterial quantification was performed from each tested surface immediately after preparation and covering. For bacterial quantification bacteria were rinsed off the surface into 10 ml Tryptic Soy Broth (TSB) with LTHTh 373r-20p (Merck, Darmstadt, Germany) and 100 μl of the suspension were streaked on COS agar plates in double determination. Colony numbers were counted for after incubation for 24 h at 36°C ± 2°C and antibacterial activity (R) was calculated as described in the ISO 22196. Confirmed antibacterial activity in the modified ISO 22196 test was defined as R> 10^2^ (at least 99% relative reduction).

### Touch transfer assay

A detailed protocol of the new assay is deposited at protocols.io (dx.doi.org/10.17504/protocols.io.i59cg96). In brief, *Enterococcus faecium* ATCC 6057 was cultivated on COS agar at 36°C ± 2°C overnight and inoculated in sterile 0.85% NaCl solution (5x10^7^ bacteria/mL). Serial tenfold dilutions of the solution were used if appropriate. 200 μl of bacterial suspensions were spread homogenously on sterilized surfaces of 5 x 5 cm ceramic tiles and dried for 1 hour under standardized conditions in a climate chamber (22°C and 50% rH) resulting in the primary contaminated surface (PCS). Uptake of bacteria from the PCS was performed with disinfected (75% Ethanol) skin of the forefinger or thumb of the test person or the forefinger or thumb covered with moistened sterile cotton gloves warn over disinfected single use nitrile gloves. Moistening and addition of organic soil load for cotton gloves mimicing the clinical situation of having organic soil matrix as a companion with bacterial burden was performed by touching COS agar for 10 sec without pressure. For the touch transfer of bacteria fingers with or without gloves were rolled like taking fingerprints without removing the finger for 10 sec on the PCS and subsequently the fingers were rolled same way on the respective sterilized surfaces for 10 sec resulting in the secondary contaminated surface (SCS). Quantitative culture of the SCS was performed immediately or after 24 h of incubation at 22°C and 50% rH in double determination or by enumeration using Replicate Organism Detection And Counting (RODAC) agar plates containing TSA with disinhibitor plus (Oxoid, Basingstoke, UK; detailed results are presented as supporting information). Statistical analysis of differences in the individual experiments were performed using t-test (R, R Foundation for Statistical Computing, Austria).

## Results

**Fig 1:**
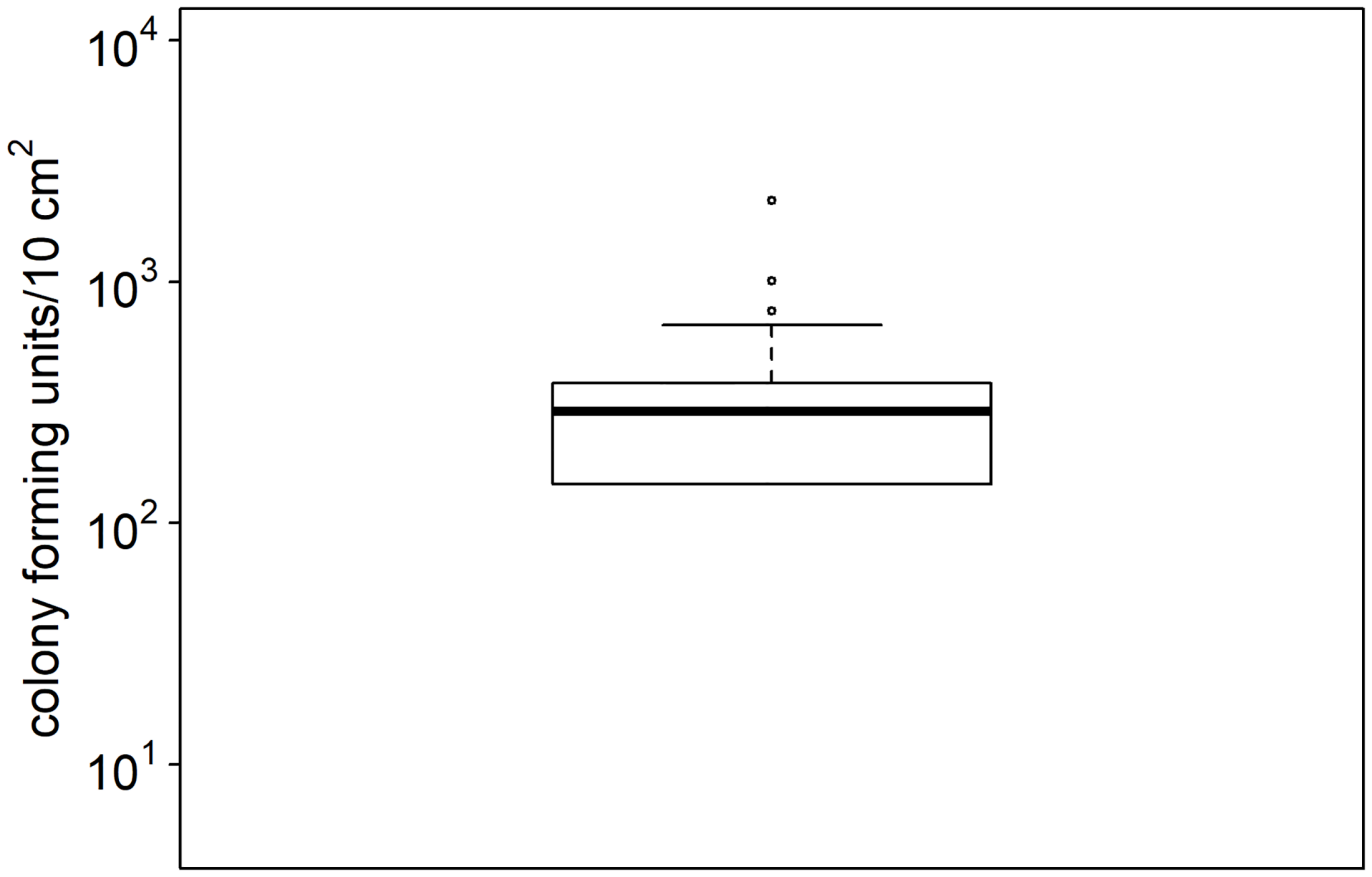
Quantification of bacteria on space bars. Space bars of computer keyboards in (n = 24) and outside (n = 23) hospitals were analyzed for the microbial burden. The boxplot displays the variation of bacterial counts (cfu) per space bar.

Quantitative culture of bacteria from space bars of 47 computer key boards revealed a median of 290 cfu/space bar (surface approximately 10 cm^2^) and a mean of 328.1 cfu/space bar with only two space bars reaching more than 10^3^ cfu (Fig 1). Bacteria detected in all environments were predominately Gram-positve species with known survival rates on dry surfaces (> 70% staphylococci, micrococci and enterococci). Therefore, a target microbial bioburden of about 10^3^ cfu but less than 10^4^ cfu was defined for SCS of 25 cm^2^ to mimic a commonly observed contamination of inanimate surfaces.

**Fig 2:**
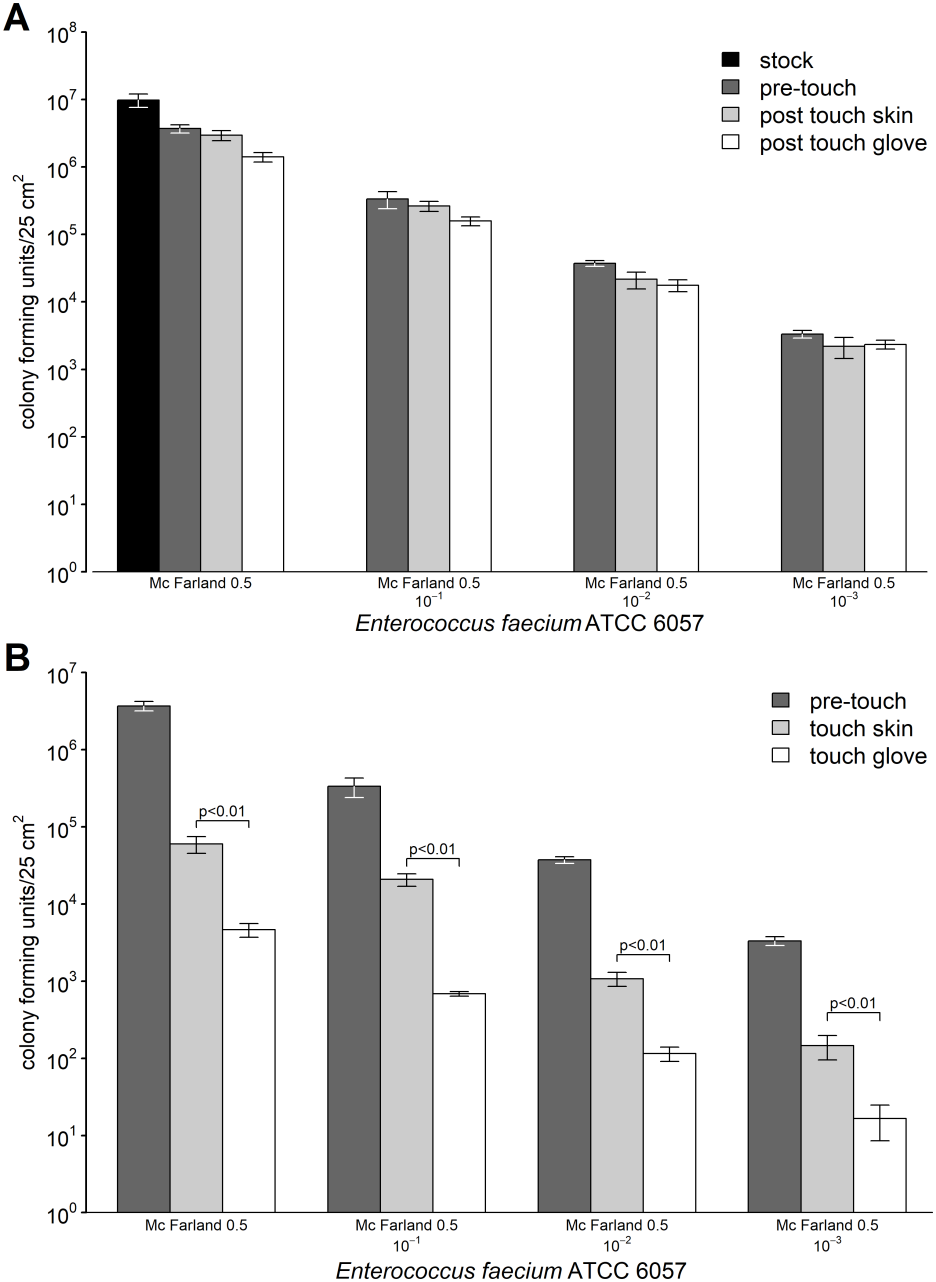
Quantification of bacteria on the PCS (A) and the SCS (B) during the touch transfer procedure. The results of three independent experiments with quantification in double determination are displayed. Serial tenfold dilutions of the stock solution (A, black, number of bacteria used to inoculate the surface of 25 cm^2^) were dried on the PCS and quantified subsequently (A, dark grey). The number of bacteria on the PCS was also quantified immediately after the uptake of bacteria by the skin of fingers (A, light grey) or by moistened sterile cotton gloves (A, white). The resulting transfer of bacteria to the SCS was quantified immediately after the touch transfer procedure by the skin of fingers (B, light grey) or by moistened sterile cotton gloves (B, white). Mean numbers of bacteria per surface (cfu/25 cm^2^) are displayed with error bars indicating the standard deviation of the respective means. Statistical significant differences between the number of transferred bacteria by touch transfer using skin or cotton gloves (B) are marked.

To be able to model touch transfer using native disinfected skin low pathogenic *Enterococcus faecium* ATCC 6057 was used displaying a low bacterial loss during desiccation and a high survival rate over time on dry surfaces [19]. During the initial desiccation process on the PCS a significant (p<0.01) loss of reculturable bacteria with 0.4 log decrease (3.7 x 10^6^/25 cm^2^ of 9.8 x 10^6^/25 cm^2^, Fig 2A) was observed. The serial dilution of bacteria had no influence on the initial loss of reculturable bacteria. The uptake of bacteria from the PCS decreased for disinfected skin corresponding with decrease of initial contamination of the PCS (7.5 x 10^5^, 7.1 x 10^4^, 1.6 x 10^4^, and 1.1 x 10^3^/25 cm^2^). The uptake of bacteria with moistened cotton gloves displayed also a decrease corresponding to the initial contamination (2.3 x 10^6^, 1.8 x 10^5^, 2.0 x 10^4^, and 9.8 x 10^2^/25 cm^2^). The deposition of bacteria on the SCS during the touch transfer procedure was significantly lower using moistened cotton gloves compared to native skin (Fig 2B). This observation could be explained by different surfaces area (cotton mesh vs. plain skin) which comes into contact with the SCS as observed by transfer of ink by fingerprints on paper using the different transfer methods (data not shown). However, the relative transfer rate remained almost stable with about tenfold difference of bacteria transferred to the SCS from PCS inoculated with tenfold serial solutions of the stock bacterial suspension. Therefore, to be able to investigate more pathogenic bacteria in the future further standardized experiments were performed using moistened cotton gloves only. For these experiments appropriate dilutions of the bacterial stock solution were used to reach the target microbial bioburden of about 10^3^ cfu but less than 10^4^ cfu.

**Fig 3:**
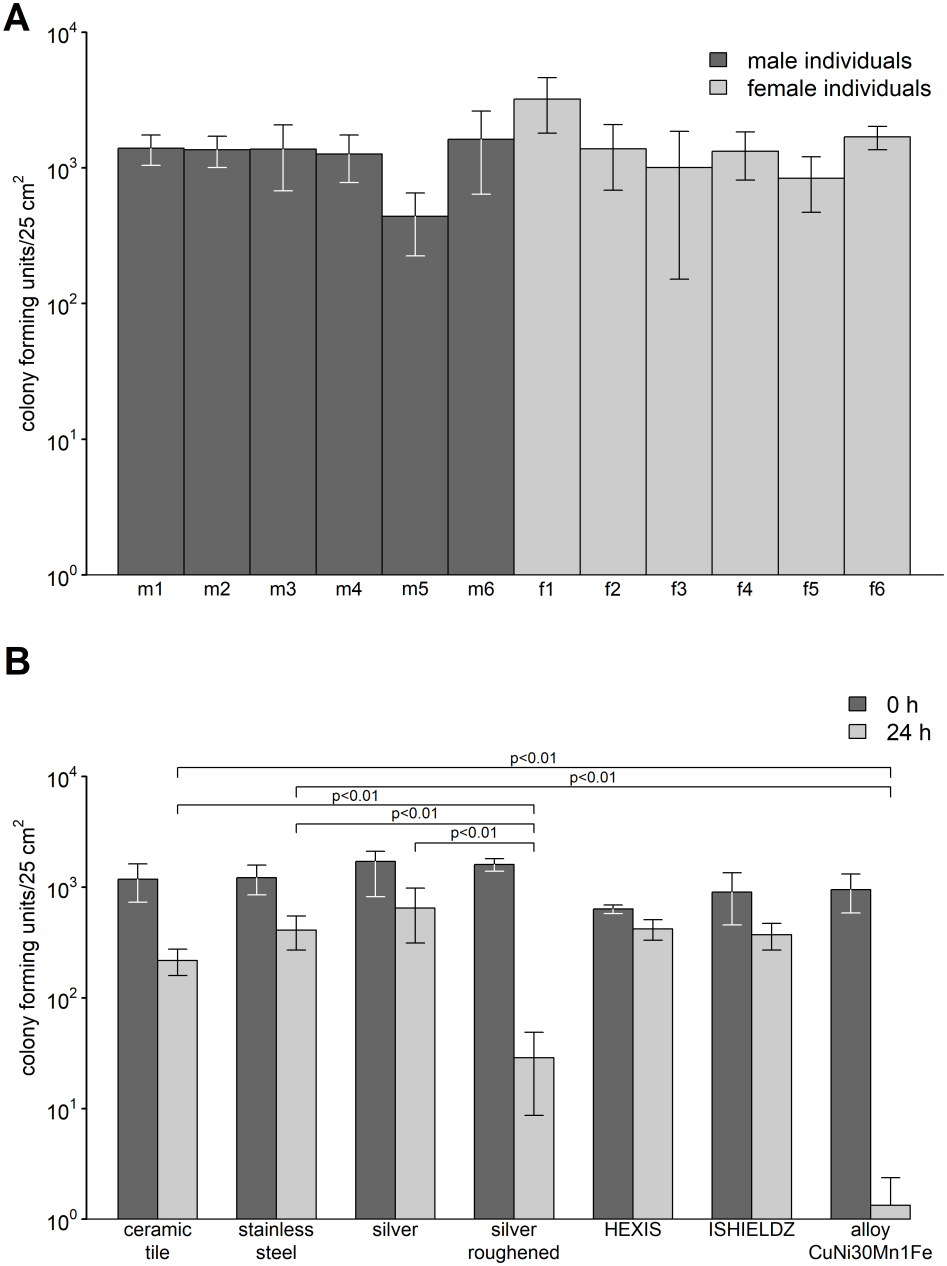
Reproducibility of the touch transfer assay (A) and investigation of potential antimicrobial surfaces (B). Six male (m1 to m6) and six female (f1 to f6) persons performed the touch transfer assay following a standard operating procedure. The results of ten independent experiments performed by each person are displayed. For the analysis of potential antimicrobial surfaces quantitative culture from SCS were performed immediately after the touch transfer (0 h, dark grey) and after 24 h of incubation at 22°C and 50% rH (24 h, light grey). The results of three independent experiments with quantification in double determination are displayed (B). Ceramic tiles and stainless steel plates were used as control. Mean numbers of bacteria per surface (cfu/25 cm^2^) are displayed with error bars indicating the standard deviation of the respective mean. Statistical significant lower bacterial counts between potential antibacterial surfaces and controls (ceramic tile and stainless steel) as well as between roughened and non-roughened pure silver are marked.

To estimate the reliability of the touch transfer assay, 12 independent investigators (6 female and 6 male persons) were asked to perform the touch transfer between PCS and SCS in ten independent experiments. All investigators except of one male person (m5) reached in average the target microbial bioburden (Fig 3A). After personal feedback to investigator m5 this person reached the target microbial bioburden in additional experiments (data not shown), indicating that the touch transfer assay can be used correctly by a wide variety of investigators.

After establishment of a standardized touch transfer procedure mimicking environmental contamination by contact to human skin several surfaces with confirmed antibacterial activity displayed in a modified ISO 22196 (JIS Z 2801) assay (data not shown) were investigated with ceramic tiles and stainless steel plates as control (Fig 3B).

Significant differences were observed for the spontaneous die off of bacteria on control surfaces. The rate of death was found to be greater on the ceramic tile after 24 h (p<0.05). Matured pure silver surfaces displayed no significant bacterial reduction compared to the control surfaces, whereas freshly roughened pure silver surfaces displayed a significant reduction compared to control surfaces as well as matured silver. Both the commercial antimicrobial films designated to supply antibacterial activity as well as non-antibacterial surfaces displayed no antibacterial activity under the ambient conditions used in the touch transfer assay. The strongest antibacterial activity in the touch transfer assay was observed for a copper alloy surface, which consistently reached values below the threshold of 2.5 cfu/cm^2^ after 24 h of incubation under dry conditions.

## Discussion

Under usual ambient air conditions of hospital rooms no condensation of humidity is expected on inanimate surfaces. In contrast, current standardized methods for the analysis of antibacterial activity of solid surfaces in general use mostly planktonic bacterial cells which are kept in thin liquid or agarose layers on tested surfaces [12;13]. Giving tribute to the contact killing mode of copper containing surfaces, individual standardized laboratory methods to demonstrate activity of copper were developed under support of the copper industry. These methods allow desiccation of bacterial inocula in different time periods [14–16]. Additionally, for copper different experimental assays were able to demonstrate antibacterial activity following a “fast desiccation” approach [17;18]. However, none of the cited laboratory methods has considered the dry transfer of bacteria from fomites simulating cross contamination so far.

Prior establishment of an assay representing the transmission, we analyzed the bioburden of frequently touched surfaces to define a commonly observed contamination rate. Thereby, we estimated a contamination of 30 cfu/cm^2^ on high touch surfaces with a maximum of about 200 cfu/cm^2^. This data display a higher contamination compared to other studies [20;21]. However, in all studies frequent transgression of the threshold proposed as a microbiologic standards for safer hospital environments (<2.5 cfu/cm^2^) were observed.

In contrast to the low average contamination observed in hospitals, current standardized methods for antimicrobial surfaces use high bacterial inocula with about 10^4^ to 10^9^ cfu/cm^2^ which do not reflect the typical environmental contamination. For the new assay we defined a target bioburden of about 40 but less than 400 cfu. This target bioburden is within the range of the observed worst case scenario and would allow identifying a bacterial killing by antimicrobial surfaces of at least one log.

To estimate the transfer rate between surfaces by human skin we analyzed the transfer of the low pathogenic *E*. *faecium* ATCC 6057 from a primary contaminated surface (PCS) by disinfected skin to a secondary contaminated surface (SCS). It could be demonstrated, that the relative uptake by skin increased from 28 to 58% with decrease of initial contamination of the PCS (Fig 2). The deposition of the bacteria to the SCS resulted in almost 10fold differences from PCS differentially contaminated, indicating a relative stable transfer by human skin. Moistened cotton gloves displayed similar uptake rates above 50% enabling the use of bacteria with higher pathogenicity in the future. The transfer to the SCS was significantly lower using cotton gloves compared to native skin which might be explained by different total surfaces which comes into contact with the SCS. However, the relative transfer rate remained also stable (tenfold differences) indicating that the designated target bioburden can be obtained by adjustment of the contamination of the PCS.

The reliability of the touch transfer assay could be demonstrated by 12 independent investigators (6 female and 6 male persons) who all hit the target microbial bioburden all in average except of one male person (Fig 3A). Personal feed back was able to adjust the transfer rate to the target bioburden with this person.

Using the novel established touch transfer assay we investigated several surfaces with confirmed antibacterial activity (Fig 3B). A significant antibacterial activity assay was observed for a copper alloy surface which resulted in almost sterile surfaces after 24 h of incubation under ambient conditions. In contrast, we were able to demonstrate, that matured pure silver surfaces displayed no significant bacterial reduction compared to control surfaces, whereas freshly roughened pure silver surfaces displayed a significant reduction. Commercially available antimicrobial films exhibit no antibacterial effect if the touch transfer assay was used. These data are corroborated by results of a recent review which clearly demonstrated that in contrast to the huge variety of antimicrobial surfaces on the market only for copper containing surfaces evidence for the impact of the surfaces on the reduction of hospital acquired infections is given [22]. For copper containing surfaces, the groups of Salgado and Schmidt were able to demonstrate significant reduction of bacterial surface contamination under commonly observed conditions, even with a significant decrease hospital acquired infections in a study blinded for control and intervention rooms [23–27]. These results under field conditions could be confirmed by our observations in the laboratory.

The lack of significant antimicrobial activity in the modified assay as developed here might have several reasons. In contrast to the original ISO 22196 assay bacteria are maintained in a liquid layer enabling diffusion of antimicrobial compounds. Additionally, a different mode of cells must be anticipated in the touch transfer assay. During the initial desiccation process on the PCS the loss of reculturable bacteria was 1.3 log on the surface (25 cm^2^) indicating transfer and contamination utilizing the preselection from survivors of desiccation, the whole package of viable cells, covered with a majority of dead cells, respectively. These organisms adapted to environmental stress might display also decreased susceptibility against antimicrobial substances as observed for antibiotics [28]. In contrast, bacterial inocula for the ISO 22196 assay are highly adapted to an artificial growth medium, which might lead to increased susceptibility. Strong differences in the bacterial inocula might also affect bacterial interaction. In bacterial populations with high density in an environment which allows diffusion of signal molecules quorum sensing mechanisms lead to differential bacterial behavior [29;30], whereas under conditions used for the touch transfer assay quorum sensing systems are not expected to be activated.

The new touch transfer assay has also some limitations which might be addressed in future research. The benefit of a typical environmental contamination (low bacterial inocula) does not allow to demonstrate the requested 99 or 99.9% reduction of microorganisms. However, the potential of surfaces to decrease a commonly observed environmental contamination below the threshold of 2.5 cfu/cm^2^ proposed as a standard for increased patient safety, can be investigated and demonstrated by the touch transfer assay.

Additionally, the touch transfer by the hand of individual investigators will always result in less standardization of the transferred cell number compared to defined volumes of liquid suspensions of microorganisms with standardized cell counts. In the future the standardization of the touch transfer assay might be increased using other materials for the transfer procedure like artificial skin [31] instead of moistened cotton gloves. Moreover a higher grade of automation for the transfer (e. g. printing tools) could facilitate the process.

In the future, assays investigating antimicrobial activity should not be adapted to the mode of action of antimicrobial compounds but should mimic commonly observed environmental conditions. Thereby, liquid based assays like the ISO 22196 should be critically be reviewed before claiming antibacterial activity for surfaces, which are in a dry state during normal use of the surface. We suggest the newly developed touch transfer assay as a new additional tool for the assessment of potential antimicrobial surfaces prior utilization in hospital environments. In the future, the transfer of bacteria in the touch transfer assay by sterile cotton gloves allows fulfilling the need to investigate also more pathogenic species like *Staphylococcus aureus*, including MRSA and other species common in hospital acquired infections.

## Supporting information

S1 Table Data for figures. Excel sheets with the detailed data of the presented figures.
(XLSX)

## References

[1] World Health Organization. Guidelines on Core Components of Infection Prevention and Control Programmes at the National and Acute Health Care Facility Level. 2016 Geneva, World Health Organization. 27977095

[2] Otter JA, Yezli S, Salkeld JA, French GL. Evidence that contaminated surfaces contribute to the transmission of hospital pathogens and an overview of strategies to address contaminated surfaces in hospital settings. Am J Infect Control 2013; 41:S6–11. doi: 10.1016/j.ajic.2012.12.004 23622751 10.1016/j.ajic.2012.12.004

[3] Dancer SJ. Importance of the environment in meticillin-resistant *Staphylococcus aureus* acquisition: the case for hospital cleaning. Lancet Infect Dis 2008; 8:101–13. doi: 10.1016/S1473-3099(07)70241-4 17974481 10.1016/S1473-3099(07)70241-4

[4] Weber DJ, Anderson D, Rutala WA. The role of the surface environment in healthcare-associated infections. Curr Opin Infect Dis 2013; 26:338–44. doi: 10.1097/QCO.0b013e3283630f04 23743816 10.1097/QCO.0b013e3283630f04

[5] Bhalla A, Pultz NJ, Gries DM, Ray AJ, Eckstein EC, Aron DC, et al Acquisition of nosocomial pathogens on hands after contact with environmental surfaces near hospitalized patients. Infect Control Hosp Epidemiol 2004; 25:164–7. doi: 10.1086/502369 14994944 10.1086/502369

[6] Otter JA, Yezli S, French GL. The role played by contaminated surfaces in the transmission of nosocomial pathogens. Infect Control Hosp Epidemiol 2011; 32:687–99. doi: 10.1086/660363 21666400 10.1086/660363

[7] Dancer SJ. How do we assess hospital cleaning? A proposal for microbiological standards for surface hygiene in hospitals. J Hosp Infect 2004; 56:10–5. 14706265 10.1016/j.jhin.2003.09.017 PMC7134512

[8] Malik RE, Cooper RA, Griffith CJ. Use of audit tools to evaluate the efficacy of cleaning systems in hospitals. Am J Infect Control 2003; 31:181–7. 12734526 10.1067/mic.2003.34

[9] Dancer SJ. Controlling hospital-acquired infection: focus on the role of the environment and new technologies for decontamination. Clin Microbiol Rev 2014; 27:665–90. doi: 10.1128/CMR.00020-14 25278571 10.1128/CMR.00020-14 PMC4187643

[10] Page K, Wilson M, Parkin IP. Antibacterial surfaces and their potential in reducing the role of inanimate environment in the incidence of hospital-aquired infections. J Med Microbiol 2009; 19:3819–31.

[11] Humphreys H. Self-disinfecting and microbiocide-impregnated surfaces and fabrics: what potential in interrupting the spread of healthcare-associated infection? Clin Infect Dis 2014; 58:848–53. doi: 10.1093/cid/cit765 24265359 10.1093/cid/cit765

[12] ASTM E2180-07 (Reapproved 2012), Standard test method fpr determining the activity of incorporated antimicrobial agent(s) in polymeric or hydrophobic materials. 2012.

[13] ISO 22196:2011, Measurement of antibacterial activity on plastics and other non-porous surfaces. 2011.

[14] US environmental protection agency. Protocol for the Evaluation of Bactericidal Activity of Hard, Non-porous Copper/Copper-Alloy Surfaces. 2015.

[15] US environmental protection agency. Test method for efficacy of copper alloy surfaces as a sanitizer. 2009.

[16] US environmental protection agency. Test Method for Residual Self-Sanitizing Activity of Copper Alloy Surfaces. 2009.

[17] Espirito SC, Taudte N, Nies DH, Grass G. Contribution of copper ion resistance to survival of Escherichia coli on metallic copper surfaces. Appl Environ Microbiol 2008; 74:977–86. doi: 10.1128/AEM.01938-07 18156321 10.1128/AEM.01938-07 PMC2258564

[18] Warnes SL, Keevil CW. Mechanism of copper surface toxicity in vancomycin-resistant enterococci following wet or dry surface contact. Appl Environ Microbiol 2011; 77:6049–59. doi: 10.1128/AEM.00597-11 21742916 10.1128/AEM.00597-11 PMC3165410

[19] Neely AN, Maley MP. Survival of enterococci and staphylococci on hospital fabrics and plastic. J Clin Microbiol 2000; 38:724–6. 10655374 10.1128/jcm.38.2.724-726.2000 PMC86187

[20] Wojgani H, Kehsa C, Cloutman-Green E, Gray C, Gant V, Klein N. Hospital door handle design and their contamination with bacteria: a real life observational study. Are we pulling against closed doors? PLoS One 2012; 7:e40171 doi: 10.1371/journal.pone.0040171 23077475 10.1371/journal.pone.0040171 PMC3471909

[21] Claro T, O'Reilly M, Daniels S, Humphreys H. Surface microbial contamination in hospitals: A pilot study on methods of sampling and the use of proposed microbiologic standards. Am J Infect Control 2015; 43:1000–2. doi: 10.1016/j.ajic.2015.05.009 26082261 10.1016/j.ajic.2015.05.009

[22] Muller MP, MacDougall C, Lim M. Antimicrobial surfaces to prevent healthcare-associated infections: a systematic review. J Hosp Infect 2016; 92:7–13. doi: 10.1016/j.jhin.2015.09.008 26601608 10.1016/j.jhin.2015.09.008

[23] Michels HT, Keevil CW, Salgado CD, Schmidt MG. From Laboratory Research to a Clinical Trial: Copper Alloy Surfaces Kill Bacteria and Reduce Hospital-Acquired Infections. HERD 2015; 9:64–79. doi: 10.1177/1937586715592650 26163568 10.1177/1937586715592650 PMC4561453

[24] Salgado CD, Sepkowitz KA, John JF, Cantey JR, Attaway HH, Freeman KD, et al Copper surfaces reduce the rate of healthcare-acquired infections in the intensive care unit. Infect Control Hosp Epidemiol 2013; 34:479–86. doi: 10.1086/670207 23571364 10.1086/670207

[25] Schmidt MG, Tuuri RE, Dharsee A, Attaway HH, Fairey SE, Borg KT, et al Antimicrobial copper alloys decreased bacteria on stethoscope surfaces. Am J Infect Control 2017. 10.1016/j.ajic.2017.01.030 28302430

[26] Schmidt MG, Attaway HH, Fairey SE, Steed LL, Michels HT, Salgado CD. Copper continuously limits the concentration of bacteria resident on bed rails within the intensive care unit. Infect Control Hosp Epidemiol 2013; 34:530–3. doi: 10.1086/670224 23571374 10.1086/670224

[27] Schmidt MG, Attaway HH, Sharpe PA, John J Jr., Sepkowitz KA, Morgan A, et al Sustained reduction of microbial burden on common hospital surfaces through introduction of copper. J Clin Microbiol 2012; 50:2217–23. doi: 10.1128/JCM.01032-12 22553242 10.1128/JCM.01032-12 PMC3405627

[28] Knobloch JK, Jäger S, Huck J, Horstkotte MA, Mack D. mecA is not involved in the sigmaB-dependent switch of the expression phenotype of methicillin resistance in *Staphylococcus epidermidis*. Antimicrob Agents Chemother 2005; 49:1216–9. doi: 10.1128/AAC.49.3.1216-1219.2005 15728932 10.1128/AAC.49.3.1216-1219.2005 PMC549230

[29] Hense BA, Kuttler C, Muller J, Rothballer M, Hartmann A, Kreft JU. Does efficiency sensing unify diffusion and quorum sensing? Nat Rev Microbiol 2007; 5:230–9. doi: 10.1038/nrmicro1600 17304251 10.1038/nrmicro1600

[30] West SA, Winzer K, Gardner A, Diggle SP. Quorum sensing and the confusion about diffusion. Trends Microbiol 2012; 20:586–94. doi: 10.1016/j.tim.2012.09.004 23084573 10.1016/j.tim.2012.09.004

[31] Arinder P, Johannesson P, Karlsson I, Borch E. Transfer and Decontamination of *S*. *aureus* in Transmission Routes Regarding Hands and Contact Surfaces. PLoS One 2016; 11:e0156390 doi: 10.1371/journal.pone.0156390 27280772 10.1371/journal.pone.0156390 PMC4900614

